# A SNOMED CT Mapping Guideline for the Local Terms Used to Document Clinical Findings and Procedures in Electronic Medical Records in South Korea: Methodological Study

**DOI:** 10.2196/46127

**Published:** 2023-04-18

**Authors:** Sumi Sung, Hyeoun-Ae Park, Hyesil Jung, Hannah Kang

**Affiliations:** 1 Biomedical Research Institute Seoul National University Hospital Seoul Republic of Korea; 2 College of Nursing Seoul National University Seoul Republic of Korea; 3 Department of Nursing Inha University Incheon Republic of Korea; 4 Kakao Healthcare Corp Seongnam-si, Gyeonggi-do Republic of Korea

**Keywords:** semantic interoperability, Systematized Nomenclature of Medicine–Clinical Terms, mapping guideline, local terms, mapping, guideline, SNOMED, nomenclature, interoperable, interoperability, terminology, medical term, health term, terminologies, ontologies

## Abstract

**Background:**

South Korea joined SNOMED International as the 39th member country. To ensure semantic interoperability, South Korea introduced SNOMED CT (Systemized Nomenclature of Medicine–Clinical Terms) in 2020. However, there is no methodology to map local Korean terms to SNOMED CT. Instead, this is performed sporadically and independently at each local medical institution. The quality of the mapping, therefore, cannot be guaranteed.

**Objective:**

This study aimed to develop and introduce a guideline to map local Korean terms to the SNOMED CT used to document clinical findings and procedures in electronic health records at health care institutions in South Korea.

**Methods:**

The guidelines were developed from December 2020 to December 2022. An extensive literature review was conducted. The overall structures and contents of the guidelines with diverse use cases were developed by referencing the existing SNOMED CT mapping guidelines, previous studies related to SNOMED CT mapping, and the experiences of the committee members. The developed guidelines were validated by a guideline review panel.

**Results:**

The SNOMED CT mapping guidelines developed in this study recommended the following 9 steps: define the purpose and scope of the map, extract terms, preprocess source terms, preprocess source terms using clinical context, select a search term, use search strategies to find SNOMED CT concepts using a browser, classify mapping correlations, validate the map, and build the final map format.

**Conclusions:**

The guidelines developed in this study can support the standardized mapping of local Korean terms into SNOMED CT. Mapping specialists can use this guideline to improve the mapping quality performed at individual local medical institutions.

## Introduction

South Korea is a leader in global information and communication technology. According to a 2021 Organization for Economic Co-operation and Development (OECD) survey of national health data infrastructure and governance, South Korea ranked second among OECD countries on data availability, maturity, and use [1]. However, data are not used fully, owing to a lack of interoperability and data security problems [2]. Interoperability is the ability of different information systems, devices, and applications to access, exchange, integrate, and cooperatively use data in a coordinated manner [3]. This occurs within and across organizational, regional, and national boundaries. Interoperability provides timely and seamless use of information and helps to globally improve the health of individuals and populations [3]. There are 4 levels of interoperability: foundational, structural, semantic, and organizational. Among them, the key strategy for ensuring semantic interoperability is the use of standard terminology that allows concepts to be represented unambiguously between the senders and receivers of information [4].

To achieve semantic interoperability, interface or local terms extracted from natural language written by a health care provider can be stored as reference terminology, such as in the SNOMED CT (Systemized Nomenclature of Medicine–Clinical Terms; SNOMED International). The stored terms with reference terminology can then be used in classification systems such as the International Classification of Diseases (ICD) for statistical purposes [2]. Health care providers in South Korea write medical records in natural language rather than using standard interface terms. Korean Standard Classification of Disease (KCD) codes are used for mortality and morbidity reports, and electronic data interchange (EDI) codes are used for national health insurance claims. Therefore, to fully use health care data, mapping terms extracted from phrases written in natural languages or using interface terms in medical records, disease classification codes, and national health insurance claim codes to reference terminologies are required.

Various efforts have mapped the terms used to document clinical findings and procedures in electronic medical records (EMRs), classification systems such as ICD 10th revision, and existing health care terminologies such as Logical Observation Identifiers Names and Codes (LOINC) and International Classification of Nursing Practice (ICNP) to the SNOMED CT in other countries [5-11]. The South Korean government, aiming to encourage the use of standard terminology in health care institutions, joined SNOMED International as the 39th member country and introduced SNOMED CT in 2020 to ensure interoperability. Subsequently, various efforts have mapped terms used to document clinical findings and procedures in EMRs or national health checkup questionnaires, classification systems including KCD-7, and EDI codes to the SNOMED CT [12-17]. Furthermore, these results are used for research purposes in the Common Data Model [17,18].

Individual medical institutions have attempted to map their terms to SNOMED CT. However, the mapping quality cannot be guaranteed due to its sporadic and independent map development. SNOMED International introduced Snap2, a tool to support mapping. However, since the tool is based on English source terms, it is difficult to apply to the Korean terms used in South Korea. This study, therefore, aimed to develop a guideline to ensure high-quality mapping of terms used to document clinical findings and procedures in the EMRs of local institutions in South Korea to SNOMED CT. The guideline focuses on a process of defining a relationship between concepts used in EMRs and the concepts of SNOMED CT [18]. The guideline does not include organizing a mapping team or reviewing existing maps. In addition, this guideline’s scope is limited to mapping to the SNOMED CT concept and excludes mapping to SNOMED CT post-coordinated expression.

## Methods

The process of developing the mapping guideline was led by a mapping guideline development committee. The committee consisted of 3 mapping experts. All committee members had SNOMED CT mapping experiences spanning more than 5 years and have conducted various national projects, such as SNOMED CT mapping of KCD-7 and EDI codes supported by the Korea Health Information Service under the Ministry of Health and Welfare in South Korea. The development of the guidelines was conducted from December 2020 to December 2022.

To develop the mapping guidelines, the committee members first developed an overall structure based on a review of the existing guidelines and their own mapping and teaching experiences. An extensive literature review was performed in PubMed, MEDLINE, and Google Scholar. The existing mapping guidelines and previous studies [19-24] were reviewed according to these criteria: (1) scope and purpose, (2) involvement of the stakeholders, (3) rigor of development, (4) clarity of presentation, and (5) applicability [25]. After the review, the existing mapping guidelines and previous studies could not be used due to the following reasons: (1) not matched in scope or purpose because of mapping SNOMED CT concepts to other classification systems such as ICD-10 or ICD-9-CM [20,21], (2) limited to automatic mapping only without any information about mapping rules [21,22], (3) lack of currency in SNOMED CT, published more than 10 years ago [20,21], and (4) no detailed information on the mapping process [23,24]. As a result, version 2.0 of the SNOMED CT-AU mapping guideline, developed by the National Electronic Health Transition Authority of Australia, also known as the Australia Digital Health Agency [19], was chosen as the framework of the guideline. The SNOMED CT-AU mapping guideline clearly describes the preprocessing process, classification and validation of mapping results, and final map structures. It matches the scope and purpose of our SNOMED CT mapping guideline and uses the most recent version of SNOMED CT as a target code among the existing guidelines. In addition, it includes rigorous mapping examples. The following sections were adopted from SNOMED CT-AU: define the purpose and scope of the map, preprocessing source terms, mapping patterns, validation, and structure of the map.

Based on the committee members’ experiences, the most difficult aspects of mapping between local terms and SNOMED CT were extracting and understanding the source terms, developing search terms, and searching the target SNOMED CT. However, the SNOMED CT-AU mapping guideline does not describe how to extract and understand source terms from EMRs, how to develop search terms, or how to search for target concepts using the SNOMED International browser. It is difficult for beginners to apply the existing SNOMED CT mapping guidelines, such as those of the SNOMED CT-AU. Therefore, the committee added 4 steps to the guideline: extract terms, preprocess source terms using clinical context, select a search term, and use search strategies to find SNOMED CT concepts using a browser.

A guideline review panel was invited to validate the guideline from May 2021 to September 2022. The review panel consisted of 3 mapping experts: a professor with more than 5 years of SNOMED CT mapping experience working at a South Korean university and 2 mapping specialists with more than 3 years of mapping experience. The first author emailed, explaining the purpose of the study, and asked to review the understandability and usability of the developed guidelines and to add more mapping examples, if possible, to the panel. In addition, 2 graduate students with no mapping experience were asked to assess whether the guideline was understandable and helpful throughout the email. These 2 processes were repeated until no more issues were identified.

## Results

### Overview

**Figure 1:**
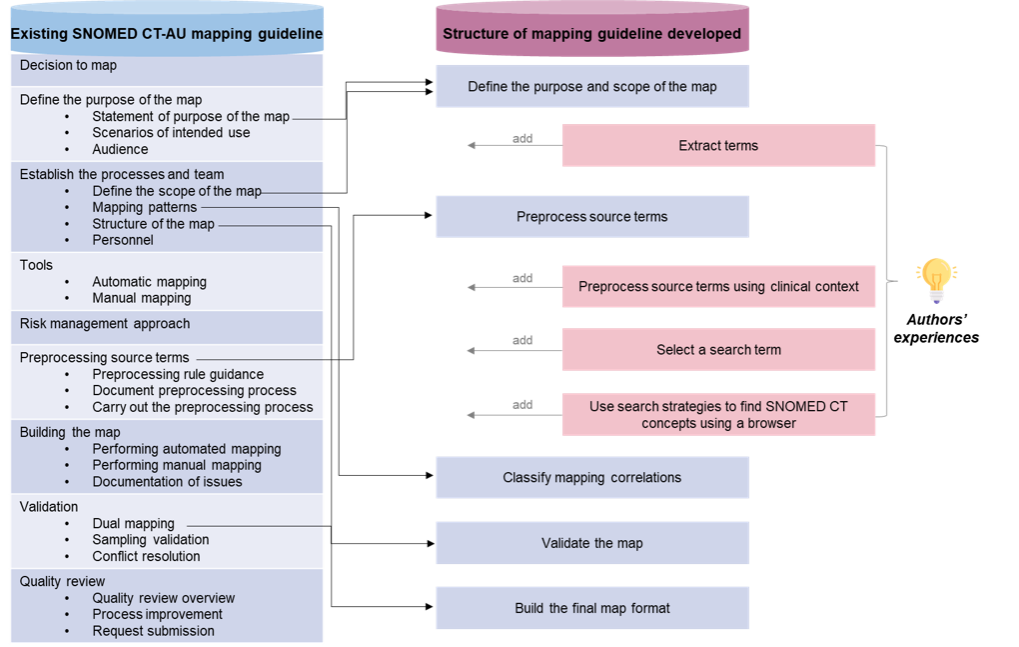
Overview of the developed guideline structure. SNOMED CT: Systemized Nomenclature of Medicine–Clinical Terms.

The SNOMED CT mapping guidelines developed in this study consisted of 9 steps, as presented in Figure 1. Step 1 defines the purpose and scope of the map. Step 2 describes how to extract source terms from EMRs. Step 3 explains how to translate the source terms into Korean, how to define abbreviations or acronyms, and how to correct spelling or punctuation errors [19]. Step 4 explains how to understand the meanings of the extracted source terms to reflect clinical contexts. Step 5 describes how to select appropriate search terms to improve mapping [26]. Step 6 describes efficient strategies to search for SNOMED CT concepts using a browser [27]. Step 7 describes how to classify the correlation between source terms and target SNOMED CT [5,7,28-31]. Step 8 explains how to validate the adequacy and accuracy of the map [19]. The final step explains how to document the final map [32]. The SNOMED CT examples used in this guideline are taken from the international edition, released on January 31, 2023.

### Step 1: Define the Purpose and Scope of the Map

**Figure 2:**
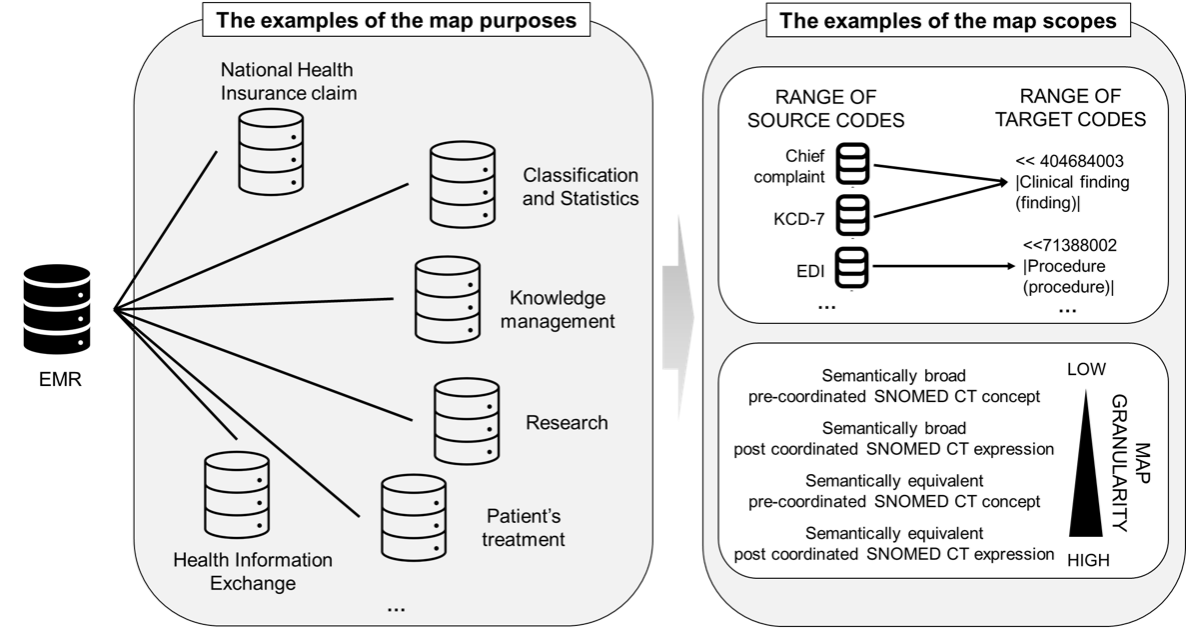
Examples of map purpose and scope. EDI: electronic data interchange; EMR: electronic medical record; KCD: Korean Classification of Disease; SNOMED CT: Systemized Nomenclature of Medicine–Clinical Terms.

To proceed with mapping, the mapping specialists must first define the purpose and scope. The purpose of the map can be for national health insurance claims, classification and statistics (eg, mortality and prevalence of specific diseases), knowledge management (eg, decision support system), health information exchange among institutions, studies (eg, Common Data Model), and patient care. Figure 2 presents examples of map purposes and scopes.

The scope of the map refers to the range of source and target codes and granularity and may differ according to its purpose. For example, if the map’s purpose is to provide prevalence statistics for specific diseases, the source codes are restricted to KCD-7 in the clinical findings domain, and the range of target codes should be SNOMED CT concepts in the clinical findings, person, or event top-level hierarchy. If the map’s purpose is for national health insurance claims, the source codes are restricted to the EDI codes in the procedure domain, and the range of target codes should be SNOMED CT concepts in the procedures’ top-level hierarchy.

Map granularity can also vary according to the purpose of the map. For example, if the map is for national health insurance claims, the local term “alcohol-related seizure,” mapped to KCD-7 code G40.5 (special epileptic syndromes), should be mapped to the abstract SNOMED CT concept 230431001 (situation-related seizures [disorder]). However, if the map is for exchanging health information among institutions, the source code should be mapped at the granular level to SNOMED CT concept 308742005 (alcohol withdrawal-induced convulsion [disorder]).

### Step 2: Extract Terms

When the purpose and scope of the map are determined, mapping specialists should extract terms from EMRs while considering where and how clinical notes are documented. Patient diagnoses can be extracted from progress notes and other similar information sources. Family and past medical histories can be extracted from progress notes and initial nursing assessment records. Surgical procedure names can be extracted from surgical notes. Evaluation procedures can be extracted from laboratory documentation.

**Figure 3:**
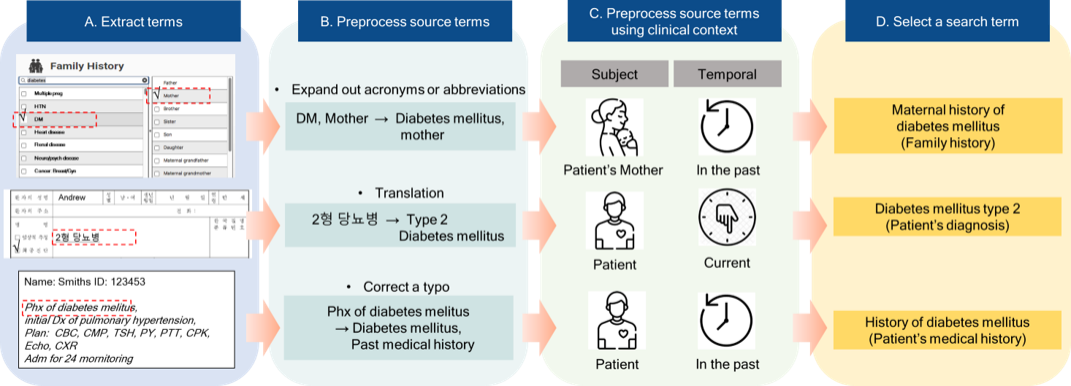
Examples of the process from term extraction to selecting a search term.

These data sources are either structured, semistructured, or unstructured medical records written in natural language; for example, as presented in step A of Figure 3, if we extract a “patient’s mother diabetes mellitus type 2,” first the interface terms from the family medical history sections in EMRs can be identified, and then the “DM” diagnosis and the “mother’s family relationship” can be extracted. The terms written in free text from the semistructured family medical history records sections of EMRs can be identified, and then the diagnosis “2형 당뇨병 (diabetes mellitus, type 2)” can be extracted. We also can identify terms from unstructured records by reading all the documents and extracting the terms written in free text, such as “Past medical history of diabetes mellitus,” which requires natural language processing (NLP).

When conducting automatic term extraction through NLP, word segmentation throughout semantic analysis is required using a corpus [33,34]. For example, the term “diabetes mellitus” should be extracted based on its meaning, not by extracting “diabetes” and “mellitus” separately. For this process to be possible, a Korean medical corpus can be used, such as the medical terminology database released by the Korean Medical Association [35] or the Korean Medical Library Engine [36]. The results of automatic terminology extraction should be reviewed manually.

Alternatively, terms can simply be extracted from code systems such as KCD-7, EDI, and local hospital codes. In these cases, it is sufficient to extract the terms mapped to the code system without duplication.

### Step 3: Preprocess the Source Terms

Preprocessing takes daily English terms as the source terms, for instance, by translating Korean terms into English. These Korean English (Konglish) terms are then translated into native English terms, with acronyms or abbreviations defined and spelling or punctuation errors corrected. If terms are written in Korean, preprocessing starts with translating the Korean terms into English. For example, “당뇨” written in Korean must be translated into “diabetes mellitus.” Konglish is often used in Korean clinical settings. It is necessary to change Konglish terms into proper English terms. In Korean clinical settings, for example, an evaluation procedure “초음파 검사” is represented by various English words such as in SONO and sonogram. SONO, or sonogram, is the image obtained using ultrasound. If it is the name of an evaluation procedure, it is, therefore, appropriate to translate it into “ultrasound.”

If terms are written in acronyms or abbreviations, which are commonly used by health care providers, preprocessing may be required to rewrite them in complete words. The acronym ASD, used in departments of pediatric cardiology, was defined as “atrial septal defect.” The abbreviation MMG used frequently in South Korea, must be rewritten to “mammography.”

Terms should be corrected when misspelled. For example, “asceding aorta dilatation” should be corrected to “ascending aorta dilatation” to obtain the correct search results. Furthermore, if terms include incorrect punctuation, it is preferable to edit the spacing of the source terms. For example, the search for the source term “DeQuervain's disease, wrist, Rt.” is missing a space, which must be added to produce “De Quervain.” Other examples are presented in step B of Figure 3.

### Step 4: Preprocess Source Terms Using Clinical Context

The terms preprocessed in the previous step can have different meanings depending on the clinical context, such as subject, temporal, or finding contexts. For example, the meaning of the preprocessed source term “diabetes mellitus” varies depending on which part of the EMR it is extracted from; it can be “diabetes mellitus,” “past medical history of diabetes mellitus,” “family history of diabetes mellitus,” or “no family history of diabetes mellitus.” “Past medical history of diabetes mellitus” means that the temporal context is in the past. “Family history of diabetes mellitus” means that the temporal context is current or in the past and that the subject is a person in the patient’s family. “No family history of diabetes mellitus” means that the temporal and subject contexts are the same as “family history of diabetes mellitus,” but the finding context is absent.

Clinical context can be inferred from the source of the structured or semistructured medical records or the text in which the source term is written. If the family history section is the source of the medical record, the subject is a family member of the patient, and the temporal context is the past or the present. If the past medical history section is the source, the subject is the patient, and the temporal context is the past or present. If the chief complaint section is the source, the subject is the patient, and the temporal context is the present. If the clinical context cannot be inferred from the structured or semistructured records, the mapping personnel should read the text in which the source term is written. Examples are presented in step C of Figure 3.

### Step 5: Select a Search Term

To search for a target SNOMED CT concept, a search term comprising keywords of the source terms that have undergone preprocessing must be selected. The search success rate is increased if the mapping specialist understands the general naming convention used by SNOMED International [26]. For example, a diagnosis or chief complaint can be expressed as “finding or morphology + body structure,” a surgery or procedure name can be expressed as “procedure + body structure,” the past medical history can be expressed as “history + disease name,” and the family medical history can be expressed as “family history + disease name.” The body structure requires “structure” to be attached to its name. Most descriptions used singular rather than plural expressions.

Stop words in NLP, which are frequently used words with restricted semantic specificity, should be excluded from search terms to improve the success rate. These stop words include articles (“a,” “an,” “the”), prepositions (“in,” “at,” “with,” “without”), conjunctions (“and,” “as”), and ambiguous adjectives or adverbs (“other,” “alone,” “single,” “side,” etc). Examples are presented in step D of Figure 3.

### Step 6: Use Search Strategies to Find SNOMED CT Concepts Using a Browser

#### Use the First 3 Characters of Words

Searches can start with the first 3 characters of search terms. With the first 3 characters, the mapping specialist can prevent search failures due to differences in the forms of various words, such as nouns, verbs, and the use of the passive voices and differences in singular and passive voices. For example, if the source term “perforated small intestine” is entered into the browser, equivalently matched concepts cannot be obtained. However, if 3 characters of each word—“per sma int”—are entered, the concept “235741002 |Perforation of small intestine (disorder)|” is matched.

When a SNOMED CT concept appears, the parent, child, and sibling concepts—higher, lower, or at the same level in the hierarchy—must be reviewed to determine if it is semantically correct.

#### Search With Synonyms of Source Terms

If the target SNOMED CT concept is not identified by search terms, synonyms should be used as search terms for the source term. The synonyms can be obtained by searching the medical terminology database released by the Korean Medical Association [34] or by searching the Korean Medical Library Engine [36]. Otherwise, the synonym can be found by including “synonym” in search terms on search engines such as Google, searching the thesaurus, reviewing websites of prestigious medical institutions, or from previous studies. In addition, conversational English terms should be changed so that different terms with equivalent meanings are also considered. For example, the conversational English phrase “removal of prostate stones” should be converted into the medically accurate phrase “removal of prostate calculus” for a successful search. Table 1 lists examples of using synonyms in a search term. If a concept is searched using synonyms, the parent, child, and sibling concepts must be reviewed in the final process to confirm whether the concept is semantically correct.

**Table 1:** Examples of using synonyms.

Source term	Search term
Removal of prostate *stones*	Removal of prostate *calculus*
Abdomen *sonogram*	*Ultrasonography* of abdomen
*Operation* of nystagmus	Nystagmus *surgery*
*Extraction* of nail	*Removal* of nail
*Correction* for rectocele	*Repair* of rectocele
Cervical mucus *test*	Cervical mucus *analysis*

#### Start With Broad or Narrow Terms

If the SNOMED CT concept is not found with synonyms of search terms, the target concept that is semantically equivalent to the source term can be searched by starting with broader or narrower terms. For example, “IUD ectopic” in the diagnosis means that an intrauterine device is in an abnormal place or position. If “intrauterine device ectopic” is entered as a search term, no search results are obtained. If “intrauterine device” (which is a broad term for a concept in the diagnosis) is searched for and the results are filtered through the “disorder” semantic tag, 16 disorder-related terms are retrieved. Among them, the “malposition of intrauterine contraceptive device” concept is semantically equivalent to the “IUD ectopic” concept. In this process, the parent, child, and sibling concepts must be reviewed to confirm whether the concept is semantically correct, as mentioned above in the final process.

#### Filter SNOMED CT Concept Using Semantic Tag

Critically, to map semantically equivalent target SNOMED CT concepts, the mapping specialist must filter results by semantic tag in the SNOMED CT browser when entering a search term. These tags are presented in Table 2. For example, if the source term “cold” in the diagnosis is entered in the browser without filters, 2 lexically matched concepts 82272006 (common cold [disorder]) and 84162001 (cold sensation quality [qualifier value]) with the same description “cold” are obtained. However, if the results are filtered through the semantic tag “disorder,” the semantically equivalent concept, 82272006 (common cold [disorder]), is immediately obtained. The diagnosis “renal cell carcinoma” must be filtered through the “disorder” semantic tag, and mapped to 702391001 (renal cell carcinoma [disorder]), not 41607009 (renal cell carcinoma [morphologic abnormality]).

**Table 2:** SNOMED CT semantic tags according to the clinical domain of the source codes.

Clinical domain of source concepts	SNOMED CT^a^ semantic tag
Chief complaint	finding
Diagnosis	disorder, finding
Past medical history, Family medical history	situation
Health-related behavior history	finding
Complication	finding
Procedure: (surgical procedure; evaluation procedure)	procedure, regime/therapy, observable entity
Pathologic diagnosis	morphologic abnormality
Medication	clinical drug, medicinal product, medicinal product form, substance

^a^SNOMED CT: Systematized Nomenclature of Medicine–Clinical Terms.

### Step 7: Classify Mapping Correlations

The mapping results can be classified according to the correlation between the source concepts and SNOMED CT concepts or according to map cardinality, depending on the number of concepts.

#### Mapping Classification According to the Correlation

According to the correlation, the mapping results can be classified as “1193548004 |Exact match between map source and map target (foundation metadata concept)|,” “1193549007 |Narrow map source to broad map target (foundation metadata concept)|,” or “1193551006 |Map source not mappable to map target (foundation metadata concept)|.” When the source term is matched to the equivalent SNOMED CT concept, the map is classified as a “1193548004 |Exact match between map source and map target (foundation metadata concept)|.” When the source term is matched to broader SNOMED CT concepts, the map is classified as a “1193549007 |Narrow map source to broad map target (foundation metadata concept)|.” When no concept broadly matches a source term, the map is classified as “1193551006 |Map source not mappable to map target (foundation metadata concept)|.”

As an example, the source term “breast cancer, upper inner quadrant” in a diagnosis is equivalently matched to 373082000 (malignant neoplasm of breast upper inner quadrant [disorder]), and the map is classified as “1193548004 |Exact match between map source and map target (foundation metadata concept)|.” Another example is that the source term “aortic valve stenosis occurred after mitral valve replacement” in the diagnosis has no equivalent SNOMED CT concept that can be equivalently mapped, so it can be mapped to the broadly matching “703223000 |Postprocedural aortic valve stenosis (disorder)|.” In this case, the map is classified as “1193549007 |Narrow map source to broad map target (foundation metadata concept)|.”

#### Classify Mapping According to Map Cardinality

If a single source code does not map to a single SNOMED CT concept, it is necessary to classify the map’s cardinality using a complex map. The mapping results can be classified as either “one to one” or “one to many” according to their cardinality. When a single source code is mapped to a single SNOMED CT concept, the map is classified as a “one to one.” When a single source code with broad or multiple meanings is mapped to multiple SNOMED CT concepts, it is classified as “one to many.” For example, the source term “right or left hemicolectomy” has 2 meanings: “right hemicolectomy” and “left hemicolectomy.” The source term is mapped to “359571009 |Right colectomy (procedure)|” and “82619000 |Left colectomy (procedure)|,” respectively, and the map is classified as “one to many.”

### Step 8: Validate the Map

The map is validated with internal and external validation. Internal validation may vary depending on how many individuals participated in the mapping and validation processes. When 2 mapping experts are involved, one maps the source terms and the other reviews the mapping results. Otherwise, both mapping experts could map the same source terms and then compare the maps constructed by each other. When more than 2 mapping experts are involved, a validation can be conducted by dividing them into 2 groups—mapping and reviewing groups. The map is deemed to be correct if the mappers and reviewers select the same results. If the maps differ, the results should be evaluated in a group discussion with the other mapper to agree on which SNOMED CT concept to use.

External validation can also be used, in which clinical or mapping experts who were not involved in the mapping process verify the validity of the mapping results. Methods for performing external validation include reviewing the sample mapping results and obtaining mapping results that were difficult to map or that were not agreed upon in an internal group discussion.

### Step 9: Build the Final Map Format

**Figure 4:**
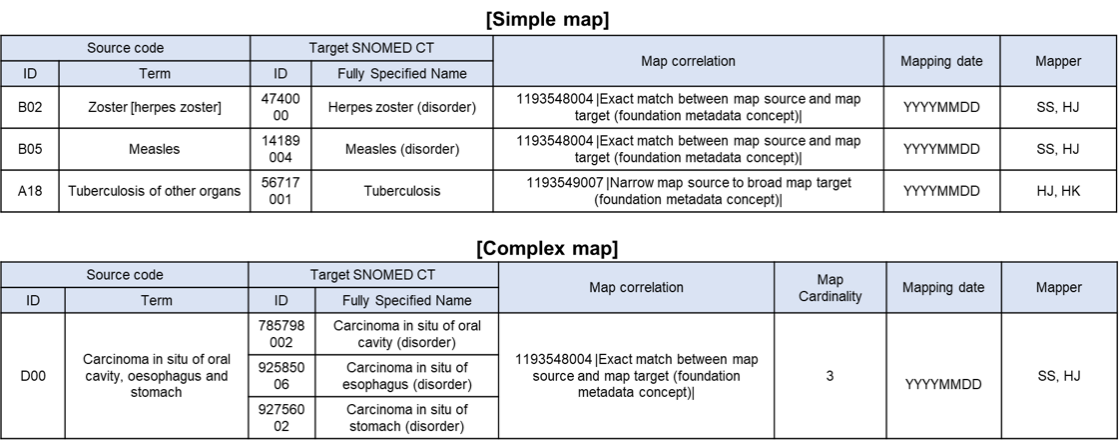
Examples of final map formats. SNOMED CT: Systemized Nomenclature of Medicine–Clinical Terms.

There are 2 types of maps—simple and complex maps. A simple map is a representation of mapping from a term in other code systems to a SNOMED CT concept; this is comprised of the source code (id, term), target SNOMED CT (id, fully specified name), and map correlation. A complex map is a representation of mapping from a term in other code systems to one or more SNOMED CT concepts; this is comprised of the source code (id, term), target SNOMED CT (id, fully specified name), map correlation, and cardinality. If map cardinality is 2 or more, rows are added to the final map. When documenting the final map, versions of source codes and SNOMED CTs, mapping dates, and mapper information must be included. Figure 4 presents examples of the mapping format.

## Discussion

### Principal Results

A SNOMED CT mapping guideline has been developed for the terms used to document clinical findings and procedures in EMRs. It was developed based on a review of previous mapping guidelines and the literature and the experiences of the authors and the map guideline development committee members. During the development of this mapping guideline, we reflected on the methods of resolving the difficulties experienced in KCD-7 and EDI code mapping, such as preprocessing the source terms and selecting search terms. We also reflected on the methods and examples identified while teaching mapping specialists working in local institutions to map the terms used to document clinical findings and procedures in EMRs to the SNOMED CT at the Korea Human Resource Development Institute of South Korea since 2019 [37]. The mapping training program was evaluated as having high satisfaction among mapping specialists with an average of 4.25 out of 5 points in 2021 (1=very low to 5=very high) and was reported as helpful in conducting mapping during work when they returned to their job with 3.75 out of 5 points in 2021 (1=not helpful to 5=very helpful). The guideline developed in this study will therefore be useful for local mapping specialists to map the terms used to document clinical findings and procedures in EMRs to the SNOMED CT. The guideline will improve the mapping quality performed at each medical institution.

The guidelines developed in this study contain detailed mapping steps from the Korean perspective, not only in the new steps such as term extraction, syntactic and semantic preprocessing, search term selection, and searching strategies in the SNOMED International browser but also in the steps adopted from previous mapping guidelines and studies [19,27,38]. For example, South Korean EMRs are written in both English and Korean. When written in Korean, translation is mandatory during preprocessing, and even when written in English, the Korean way of expressing a medical concept in English differs from that in English-speaking countries, so additional preprocessing is also required. Since Korean mapping specialists are limited by their knowledge of English synonyms, examples of synonyms were included to make the guideline easier to understand. The guideline can, therefore, be applied in local medical institutions to map Korean terms used to document clinical findings and procedures in EMRs to SNOMED CT. They can also be used to develop SNOMED CT mapping guidelines in other countries.

### Limitations

This study had some limitations. Since the guideline focused on specific clinical domains, other clinical domains, such as medicine, were not included. Future studies are required on the development of mapping guidelines for terms used to document other clinical domains. In addition, this guideline does not include a guideline for mapping to SNOMED CT post-coordinated expression. The post-coordinated expressions frequently used in South Korea can be added as a new concept in the Korean extension of SNOMED CT.

### Conclusions

This study developed a SNOMED CT mapping guideline for the terms used to document clinical findings and procedures in EMRs at local institutions in South Korea. The guideline was based on existing mapping guidelines, the findings of previous studies, and the mapping and teaching experiences of the authors. The mapping guideline developed in this study consisted of the following nine steps: (1) define the purpose and scope of the map, (2) extract terms, (3) preprocess source terms, (4) preprocess source terms using clinical context, (5) select a search term, (6) use search strategies to find SNOMED CT concepts using a browser, (7) classify mapping correlations, (8) validate the map, and (9) build the final map format. The new guideline can be published on the website of the Korea Health Information Service. The guideline can be applied to local medical institutions when mapping Korean terms used to document clinical findings and procedures in EMRs to SNOMED CT. It will also support local medical institutions in standardizing their local code systems using SNOMED CT. Ultimately, the data quality of each local medical institution will be improved, allowing the data to be fully used in clinical decision support systems, health information exchange, and clinical research.

## Abbreviations

EDI: electronic data interchange
EMR: electronic medical record
ICD: International Classification of Diseases
ICNP: International Classification of Nursing Practice
KCD: Korean Standard Classification of Disease
LOINC: Logical Observation Identifiers Names and Codes
NLP: natural language processing
OECD: Organization for Economic Co-operation and Development
SNOMED CT: Systemized Nomenclature of Medicine–Clinical Terms
